# Topical mitomycin in endoscopic-assisted probing for the treatment of congenital nasolacrimal duct obstruction in older children

**DOI:** 10.5935/0004-2749.20200062

**Published:** 2020

**Authors:** Alicia Galindo-Ferreiro, Rajiv Khandekar, Patricia Mitiko Akaishi, Antonio Augusto Velasco Cruz, Alberto Galvez-Ruiz, Angela Dolmetsch, Silvana Artioli Schellini

**Affiliations:** 1 King Khaled Eye Specialist Hospital, Riyadh, Saudi Arabia; 2 Department of Ophthalmology, Rio Hortega University Hospital, Valladolid, Spain; 3 Department of Ophthalmology, Otorhinolaryngology and Head and Neck Surgery, Faculdade de Medicina de Ribeirão Preto, Universidade de São Paulo, Ribeirão Preto, SP, Brazil; 4 Clinica de Oftalmología de Cali, Cali, Colômbia; 5 Department of Ophthalmology, Faculdade de Medicina de Botucatu, Universidade Estadual Paulista “Prof. Júlio de Mesquita Filho”, Botucatu, SP, Brazil

**Keywords:** Mitomycin/therapeutic use, Endoscopy, Lacrimal duct obstruction/congenital, Nasolacrimal duct/drug effects, Mitomicina/uso terapêutico, Endoscopia, Obstrução do ducto lacrimal/congênito, Ducto nasolacrimal/efeito dos fármacos

## Abstract

**Purpose:**

Mitomycin C has been used in ophthalmic surgery to mitigate postoperative scarring. However, the outcomes of endoscopic-assisted probing for the treatment of congenital nasolacrimal duct obstruction with adjunctive mitomycin C in children remain unknown. Our study was aimed to evaluate the efficacy and safety of adjunctive application of mitomycin C after endoscopic-assisted probing for the treatment of congenital nasolacrimal duct obstruction in children.

**Methods:**

This is a retrospective chart review performed in a tertiary eye care hospital involving children with congenital nasolacrimal duct obstruction, who underwent endoscopic-assisted probing from October 2013 to August 2015. We compared children who underwent endoscopic-assisted probing with mitomycin C (mitomycin C group) versus others who underwent endoscopic-assisted probing without mitomycin C (endoscopic-assisted probing group). The mitomycin C group received 0.2 mg/ml within 4 min to the nasolacrimal duct ostium using a cotton tip applicator immediately after probing. Probing was considered successful when patient complaints of tearing were reduced or the results of the dye disappearance test were normal. Demographic data, clinical features, and intraoperative and postoperative variables were correlated to the success rate.

**Results:**

The study sample comprised 68 lacrimal vies. The majority of children had bilateral obstruction and no previous history of probing. The mean age of the patients was approximately 4 years. Most obstructions were considered complex. The success rates were high in both groups (p>0.05). There were no adverse events related to the use of mitomycin C (p>0.05).

**Conclusions:**

Although mitomycin C has no adverse effects when applied to the opening of the nasolacrimal duct, its use after lacrimal probing for the treatment of congenital nasolacrimal duct obstruction does not improve the chance of success.

## Abstract (pt)

**Objetivo:**

A mitomicina C tem sido usada em cirurgia oftálmica para reduzir cicatrizes pós-operatórias. Entretanto, os resultados da sondagem endoscópica assistida para o tratamento da obstrução congênita do ducto nasolacrimal com mitomicina C adjuvante em crianças permanecem desconhecidos. Nosso estudo teve como objetivo avaliar a eficácia e a segurança da aplicação da mitomicina C após a sondagem endoscópica assis tida para o tratamento da obstrução congênita do ducto nasolacrimal em crianças. **M**é**todos:** Trata-se de uma revisão retrospectiva de prontuários, realizads em um hospital terciário de oftalmologia, envolvendo crianças com obstrução congênita do ducto nasolacrimal, submetidas à sondagem endoscópica de Outubro de 2013 a Agosto de 2015. Comparamos crianças submetidas à sondagem endoscópica com mitomicina C (grupo mitomicina C) versus outros que foram submetidos à sondagem endoscópica sem mitomicina C (grupo de sondagem endoscópica). O grupo mitomicina C recebeu 0,2 mg/ml em 4 min para o óstio do ducto nasolacrimal usando um aplicador de ponta de algodão imediatamente após a sondagem. A sondagem foi considerada bem-sucedida quando as queixas de lacrimejamento dos pacientes foram reduzidas ou os resultados do teste de desaparecimento do corante foram normais. Dados demográficos, sinais clínicos, variáveis intra e pós-operatórias foram correlacionados com a taxa de sucesso.

**Resultados:**

A amostra do estudo foi composta por 68 vias lacrimais. A maioria das crianças apresentava obstrução bilateral e sem histórico prévio de sondagem. A média de idade dos pacientes era de aproximadamente 4 anos. A maioria das obstruções foi considerada complexa. As taxas de sucesso foram altas nos dois grupos (p>0.05). Não houve efeitos adversos rel acionados ao uso da mitomicina C (p>0.05).

**Conclusões:**

Apesar a mitomicina C não tenha efeitos adversos quando aplicada à abertura do ducto nasolacrimal, seu uso após sondagem lacrimal no tratamento da obstrução congênita do ducto nasolacrimal não melhora a chance de sucesso.

## INTRODUCTION

Mitomycin C (MMC) is an antineoplastic agent that inhibits the synthesis of DNA, cellular RNA, and protein by blocking collagen synthesis^(1)^. MMC has been extensively studied, and its use for ophthalmic indications (e.g., adjunctive treatment for pterygium^(2)^ and glaucoma surgery) is considered safe and effective^(3)^.

In addition, MMC has been used as an adjunctive treatment in lacrimal drainage surgery^(4-7)^. Intraoperative nasal application of MMC appears to be a safe adjuvant that may reduce the closure of the ostium created between the sac and the nasal cavity^(8)^. This application of MMC may increase the success rate of primary surgery or revision of endoscopic dacryocystorhinostomy (ENDO-DCR)^(9)^ or external DCR^(8)^. It has been shown that MMC improves the success rate of probing in adults^(10-13)^.

In children, MMC is useful as an adjunctive treatment for choanal atresia repair^(14)^, management of laryngeal and tracheal stenosis^(15)^, and prevention of recurrence of caustic esophageal strictures^(16)^. Improved outcomes have been reported in children with congenital glaucoma who have received adjunctive MMC during surgery^(17-19)^.

ENDO-DCR combined with MMC in children is a safe and effective option and is associated with high success rates^(20)^. However, there are no studies investigating endoscopic-assisted probing (EAP) for the treatment of con genital nasolacrimal duct obstruction (CNLDO) in older children using MMC as an adjunctive treatment. Thus, the present study was performed to compare the outcome and safety of EAP with and without adjunctive MMC in the treatment of CNLDO in children.

## METHODS

This retrospective study compared the success rate of EAP with adjunctive MMC (MMC group) versus that of EAP without MMC (EAP group). The study population comprised children aged <12 years with CNLDO, who underwent EAP at a tertiary eye hospital in central Saudi Arabia from October 2013 to August 2015. The study protocol was approved by the Institutional Review Board of the King Khaled Eye Specialist Hospital Institutional Research Board (Riyadh, Saudi Arabia). Owing to the nature of the study, informed consent was not required.

A chart review was performed to collect patient data. CNLDO was suspected based on the symptoms of epiphora with or without discharge since birth or onset within the first 15 days of life. The diagnosis was confirmed based on excessive lacrimal meniscus, a positive fluor escein dye disappearance test, and positive reflux at compression of the lacrimal sac. Patients with CNLDO and at least one completely patent punctum and canaliculus, patent common canaliculus, with or without previous failed probing, and previous acute or chronic dacryocystitis were included. Exclusion criteria were previous DCR, with complete obstruction of the upper and lower canaliculus, bony obstruction of the nasolacrimal duct, agenesis of both lacrimal puncta, nasolacrimal obstruction secondary to trauma, tumors, or systemic diseases (e.g., sarcoidosis and Wegener’s).

### Classification of CNLDO

For the purpose of this study, CNLDO was classified as simple/membranous and complex/firm based on previous studies^(21,22)^ and endoscopic evaluation of the opening of the lacrimal system under the inferior cornetus. A simple obstruction indicated minimal resistance to the probe until it entered the lower aspect of the nasolacrimal duct, at which point a thin membrane was encountered in the region of the valve of Hasner. Following perforation of this thin membrane, the probe could be repeatedly passed without any evidence of obstruction. Complex CNDLO indicated obstructions without passage of the probe through the valve of Hasner, or with endonasal causes causing obstruction (e.g., large impacted turbinate to the lateral wall, a thick membrane, cysts, or granulomas) in the region of the valve of Hasner.

### Probing procedure

Probing procedures were performed under general anesthesia and assisted with a nasal endoscope. EAP was performed by initially instilling two drops of xylo metazoline 0.05% (nasal spray Pediatric Otrivine^®^; Novartis AG, Basel, Switzerland) or diluted adrenaline (1 ampule in 10 cc physiologic saline) in each nostril 15 min prior to the initiation of probing. Nasal packing was placed in the nostril with 5 cc of diluted adrenaline in physiologic saline. Packing was attempted under the lower turbinate, maintained in place undisturbed for 10 min, and subsequently removed. The upper and lower lacrimal puncta were enlarged using a dilator. Nasal evaluation was performed with an endoscope (Tricam SL Endoscope; KARL STORZ GmbH and Co. KG, Tuttlingen, Germany) while injecting dilute fluorescein in the upper canaliculus. In the presence of complete obstruction (or atresia) or relative stenosis of the lower end of the nasolacrimal duct, a 0-00 Bowman lacrimal probe (KARL STORZ GmbH and Co. KG) was directed nasally and advanced gently until the obstruction was encountered. At this point, the 2.7 mm diameter endoscope, with a 0º or 30º viewing angle was introduced in the nostril, and the inferior turbinate was lifted until the area of the opening of the nasolacrimal duct. In patients with shallow space between the inferior turbinate and the lateral wall of the nose, the turbinate was fractured with a Freer elevator. The probe was pushed until the membranous stop prevented further advancement of the probe tip, and this anatomy could be visualized with the endoscope. Following the detection of a “bony stop” in the nasolacrimal duct, the patient was excluded from the present study and scheduled for DCR. While visualizing the probe in the valve of Hasner with the endoscope, the probe was pushed through the membranous obstruction. In case of enlargement, the sac was squeezed to release the accumulated mucus into the nose. The opening of the valve of Hasner was observed to note flaps or redundant mucosa, which could act as a trapdoor. Flap or membranes were excised using curved or straight micro ear forceps. The nasolacrimal duct was irrigated with diluted fluorescein in saline solution through the upper or lower punctum to verify free flow of fluid into the nose, ensuring a successful procedure^(22)^. In patients with bilateral obstruction, the procedure was immediately repeated (same day) on the other side.

### Application of MMC

A sterile cotton tip applicator (Q-Tip) was soaked in MMC (Mitomycin for injection USP 5 mg, Accord; Intas Pharmaceuticals Ltd., Ahmedabad, India) at a concentration of 0.2 mg/ml. The Q-tip was placed in the inferior meatus at the nasolacrimal duct ostium site for 4 min and subsequently discarded.

### Stent intubation

In patients who underwent EAP for partial canalicular stenosis or partial obstruction of the upper end of the nasolacrimal duct, a Crawford Bicanalicular Intubation (FCI Ophthalmics, Pembroke, MA, USA) was placed. If only one canaliculus was patent, the Minimonoka (FCI Ophthalmics) was inserted.

After probing, topical neomycin, bacitracin, and dexamethasone (Maxitrol; Alcon Inc., Fort Worth, TX, USA) were administered four times per day for 10 days. Fluticasone pediatric nasal spray (Avamys^®^; GlaxoSmithKline, London, UK) was administered twice daily for 10 days in the nostril that underwent probing. Postoperative follow-up was performed at 1, 3, 6, and 12 months postoperatively, and the patency of the lacrimal drainage system was assessed.

### Main outcome measures

Success was based on clinical signs, including an asymptomatic patient or improvement in tearing after probing, and objective patency of drainage using the fluorescein dye disappearance test. The duration of the follow-up was calculated as the date of follow-up subtracted from the date of probing.

### Statistical analysis

The sample size was calculated using the Open epi software^(23)^, considering that this was a retrospective cohort study with two arms and assuming that the success rate in older children with CNLDO who underwent EAP with MMC as an adjuvant therapy was ≥85%^(22)^. It was estimated that ≥30 lacrimal vies in each group were necessary to achieve 90% success rate and 95% confidence interval (CI) in a cohort study with a 1:1 ratio. Children were not randomly allocated to the groups owing to the retrospective nature of the study.

The data were collected on an Excel spreadsheet (Microsoft Corp., Redmond, WA, USA) and transferred for analysis to the Statistical Package for Social Studies (SPSS version 19.0; IBM Corp., Armonk, NY, USA). Based on the type and distribution of the demographic variables, they are presented as frequency percentage proportion or mean ± standard deviation or median (interquartile range). The success rates for both methods and the corresponding 95% CI are reported in this study.

The success rates of both methods were tested using the chi-squared test or Fisher’s exact test, considering the patient age at the time of the procedure. A p<0.05 indicated statistical significance.

## RESULTS

The study sample comprised 30 and 38 lacrimal vies in the MMC and EAP groups, respectively. Comparison of patient demographics and clinical findings of both groups are presented in table 1. The majority of patients were females, and the majority of obstructions were bilateral and complex (Table 1). The median duration of the follow-up was 5.7 months (25% quartile; 2.9 months) for the MMC group and 3.2 months (25% quartile; 1.5 months) for the EAP group (Kruskal-Wallis test: p=0.08).

**Table 1:** Profile of patients treated with endoscopic-assisted probing to manage congenital nasolacrimal duct obstruction with and without adjunctive treatment with mitomycin C

Number		MMC group (n=30)		EAP group (n=38)		
		Number	%	Number	%	
Sex	Male Female	14 16	46.7 53.3	11 27S	28.9 71.1	OR=2.1 95% CI: 0.5-5.8 P=0.1
Side	Right Left	18 12	60.0 40.0	22 16	57.9 42.1	OR=1.1 95% Cl: 0.4-2.9 P=0.9
Laterality	Unilateral Bilateral	6 24	20.0 80.0	21 17	55.3 44.7	OR=0.2 95% Cl: 0.1-0.5 P=0.001
History of probing	Yes No	4 26	13.3 86.7	5 33	13.2 86.8	OR=1.0 95% Cl: 0.2-4.2 P=0.9
Main complaints	Watering Discharge	21 9	70.0 30.0	23 15	60.5 39.5	OR=1.5 95% Cl: 0.6-4.2 P=0.4
Type of obstruction	Simple Complex	7 23	23.3 76.7	11 27	28.9 71.1	OR=0.7 95% Cl: 0.2-2.2 P=0.6
Stent used	Yes No	5 25	16.7 83.3	14 24	36.8 63.2	OR=0.3 95% Cl: 0.1-1.1 P=0.07
Age (months)	Mean Sdv	55.8 32.5		52.2 29.0		Diff of mean -3.4 95% Cl: -18.8 to 11.5 P=0.63

EAP with MMC (MMC group), EAP without MMC (EAP group).

Comparisons of the outcomes of probing between the groups are presented in table 2. In the MMC group, all 30 lacrimal vies (100%) were successfully treated. In the EAP group, success was reported for 34 lacrimal vies (89.5%). The difference was not statistically significant (p=0.09).

**Table 2:** Success rate of endoscopic-assisted probing for the treatment of congenital nasolacrimal duct obstruction with and without the use of mitomycin C

	MMC group (n=30)		EAP group (n = 38)	
	Number	%	Number	%
Success	**30**	**100**	34	**89.5 P=0.09**
Failure	**0**	**0.0**	4	**10.5**

EAP with MMC (MMC group), EAP without MMC (EAP group).

However, the success rate between the groups was significantly influenced by bilateral or unilateral nature of CNLDO (p=0.008) (Table 3). Demographics, previous probing, and type of obstruction did not affect the success rates (Table 3).

**Table 3:** Successful outcomes of endoscopy-assisted probing for the treatment of congenital nasolacrimal duct obstruction with and without the use of mitomycin C and its determinants

Number		Success with the use of mitomycin C (n=30)		Success without using mitomycin C (n=34)		
		Number	%	Number	%	
Sex	Male	8	33.3	**6**	26.1	OR=1.4
	Female	16	66.7	17	73.9	95% Cl: 0.4-5.0
						P=0.6
Side involved	Right	18	60.0	20	58.8	OR=1.1
	Left	12	40.0	14	41.2	95% Cl: 0.4-2.8
						P=0.9
Laterality	Unilateral	6	20.0	18	52.9	OR=0.2
	Bilateral	24	80.0	16	47.1	95% Cl: 0.1-0.7
						P=0.008
History of probing	Yes	4	13.3	4	11.8	OR=1.2
	No	26	86.7	30	88.2	95% Cl: 0.3-5.1
						P=0.8
Type of obstruction	Simple	7	23.3	11	32.4	OR=0.6
	Complex	23	76.7	23	67.6	95% Cl: 0.2-1.9
						P=0.4
Stent used	Yes	5	16.7	13	38.2	OR=0.3
	No	25	83.3	21	61.8	95% Cl: 0.1-1.1
						P=0.06
Age (month)	Mean		55.9	51.4		Diff of mean -4.5
	Sdv		30.9	24.8		95% Cl: -21.0 to 12.0
						P=0.6

The high success rate and small sample sizes of the subgroups precluded regression analysis of the interaction between significantly associated factors and success of probing.

## DISCUSSION

The present study investigated whether application of MMC at the ostium of the nasolacrimal duct improves the success rates of EAP for the treatment of CNLDO. In the current study, the sample was composed of children with a mean age of approximately 4 years (55.8 and 52.2 months in the MMC and EAP groups, respectively). Additionally, 80% and >70% of the patients had bilateral CNLDO and complex obstructions, respectively. The success rates of EAP for CNLDO ranged from 70% to 100%. However, older children, as well as bilateral and complex obstruction^(21,22,24-30)^ (all characteristics presented in the patients enrolled in our study) were linked to lower success. Hence, we elected to use adjunctive treatment to improve outcomes.

Our choice of MMC was based on its antiproliferative and anti-inflammatory properties, such reduction of inflammation and fibrosis, reduced vascular ingrowth, and mitigation of scar formation. The patients were old with the obstruction since birth. Owing to this characteristic, we speculated that the inflammatory component in the lacrimal ducts may be important to justify the use of MMC. After DCR, cicatricial closure of the ostium is a common cause of failure. The application of MMC may increase the success rate of surgery^(4,6,31)^.

We decided to apply 0.2 mg/ml MMC for 4 min. The duration and concentration of MMC remain debatable, with reported concentrations ranging from 0.2 mg/ml to 0.5 mg/ml and durations between 2 min and 48 h^(7,31)^. Of note, the minimum effective concentration is 0.2 mg/ml for 3 min^(32)^. The use of other concentrations or duration of application may have resulted in different outcomes^(32)^.

The central role of fibroblast proliferation and extracellular matrix production after probing is well established^(12,33)^. Additionally, intraoperative use of MMC pre vents excessive shrinkage in ostium size, as well as common canalicular obstruction^(6)^.

In our study, there was a 100% and 89.5% success rate in the MMC and EAP group (without MMC), respectively. However, this difference was not statistically significant, indicating that both procedures provide good outcomes (p>0.05). The same observation is noted when MMC is applied after ENDO-DCR for the treatment of obstructions in adults^(34,35)^.

The factors associated with success in our study were application of MMC, laterality, and placement of stents. However, the small sample sizes of the subgroups warrant cautious interpretation of the present findings.

The lack of postoperative side effects and adverse events in our study indicates that the application of MMC in the nostrils is safe. This was also observed when MMC was used in ENDO-DCR, including a lack of abnormal nasal bleeding, mucosal necrosis, or infection^(5,6,34)^.

The antiangiogenic activity of MMC has been associated with some systemic toxicity and side effects, including bone marrow suppression and hepatorenal disease^(9)^. The side effects associated with the application of MMC to the ocular surface include changes in wound healing and corneal ulcers^(9)^. However, we did not observe any systemic or local side effects in our patients.

This study was characterized by some limitations. Despite having an adequate sample to observe the effect of MMC in the treatment of CNLDO in older children, the high success rates reported in both groups may have influenced the analysis. Hence, uncertainty remains regarding the determinants of failure. Additionally, the interaction between stents, unilateral or bilateral di sease, and the use of MMC could not be evaluated due to the small sample sizes of the subgroups. Further studies using postoperative nasal endoscopy and systemic analysis may provide additional information on this important topic.

In conclusion, MMC is not associated with adverse effects when applied to the opening of the nasolacrimal duct. However, its use as an adjunctive to lacrimal probing for the treatment of CNLDO does not improve the chance of success. Prospective, randomized studies are required to confirm the observations of the present study and investigate the influence of factors associated with successful outcomes.
